# Supplementary material for: Protective Effects of 3′-Epilutein and 3′-Oxolutein against Glutamate-Induced Neuronal Damage
Source: Int J Mol Sci. 2023 Jul 26;24(15):12008. doi: 10.3390/ijms241512008 (PMC10418699; doi:10.3390/ijms241512008)
Supplement: Supplementary file 1 [file ijms-24-12008-s001.zip › ijms-2510003-supplementary.pdf]

Supplementary Materials

Protective effects of 3′-epilutein and 3′-oxolutein against glutamate-induced neuronal damage

Ramóna Pap [1], Edina Pandur [1], Gergely Jánosa [1], Katalin Sipos [1], Ferenc Rómeó Fritz [1], Tamás Nagy [2], Attila Agócs [3] and József Deli [3,4*]

[1] Department of Pharmaceutical Biology, Faculty of Pharmacy, University of Pécs, Rókus u. 2, H-7624 Pécs, Hungary
[2] Department of Laboratory Medicine, Faculty of Medical Sciences, University of Pécs, Ifjúság út 13, H-7624 Pécs, Hungary
[3] Department of Biochemistry and Medical Chemistry, Medical School, University of Pécs, Szigeti út 12, H-7624 Pécs, Hungary
[4] Department of Pharmacognosy, Faculty of Pharmacy, University of Pécs, Rókus u. 2, H-7624 Pécs, Hungary
* Correspondence: jozsef.deli@aok.pte.hu

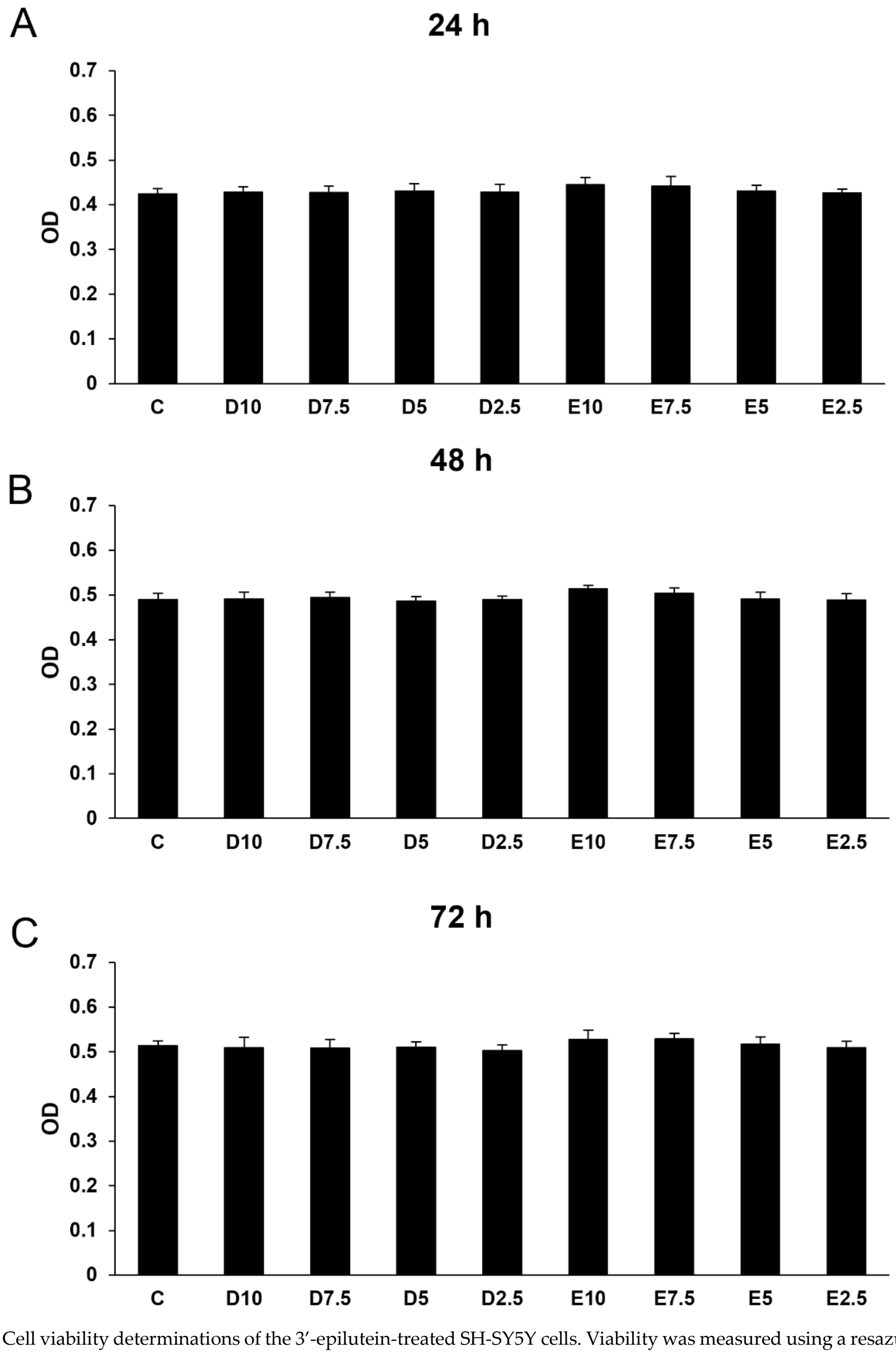

Figure S1. Cell viability determinations of the 3′-epilutein-treated SH-SY5Y cells. Viability was measured using a resazurin-based cell viability assay after 3′-epilutein treatments. The mean optical density of the wells indicates the viable cells from four independent experiments, each measured in triplicate. Viability is expressed as a percentile of the total cell number of the appropriate DMSO-treated control cells. (A) Cell viability of 3′-epilutein and DMSO-treated SH-SY5Y cells at 24 h. (B) Cell viability of 3′-epilutein and DMSO-treated SH-SY5Y cells at 48 h. (C) Cell viability of 3′-epilutein and DMSO-treated SH-SY5Y cells at 72 h. The bars represent mean values and error bars represent the standard deviation (± SD) for four independent experiments (n=4). Abbreviations of treatments: C-absolute control; DMSO controls: D2.5-DMSO equivalent to 2.5 ng/μL of 3′-epilutein; D5-DMSO equivalent to 5 ng/μL of 3′-epilutein; D7.5-DMSO equivalent to 7.5 ng/μL of 3′-epilutein; D10-DMSO equivalent to 10 ng/μL of 3′-epilutein; E2.5- 2.5 ng/μL of 3′-epilutein; E5- 5 ng/μL of 3′-epilutein; E7.5- 7.5 ng/μL of 3′-epilutein; E10- 10 ng/μL of 3′-epilutein; OD-optical density.

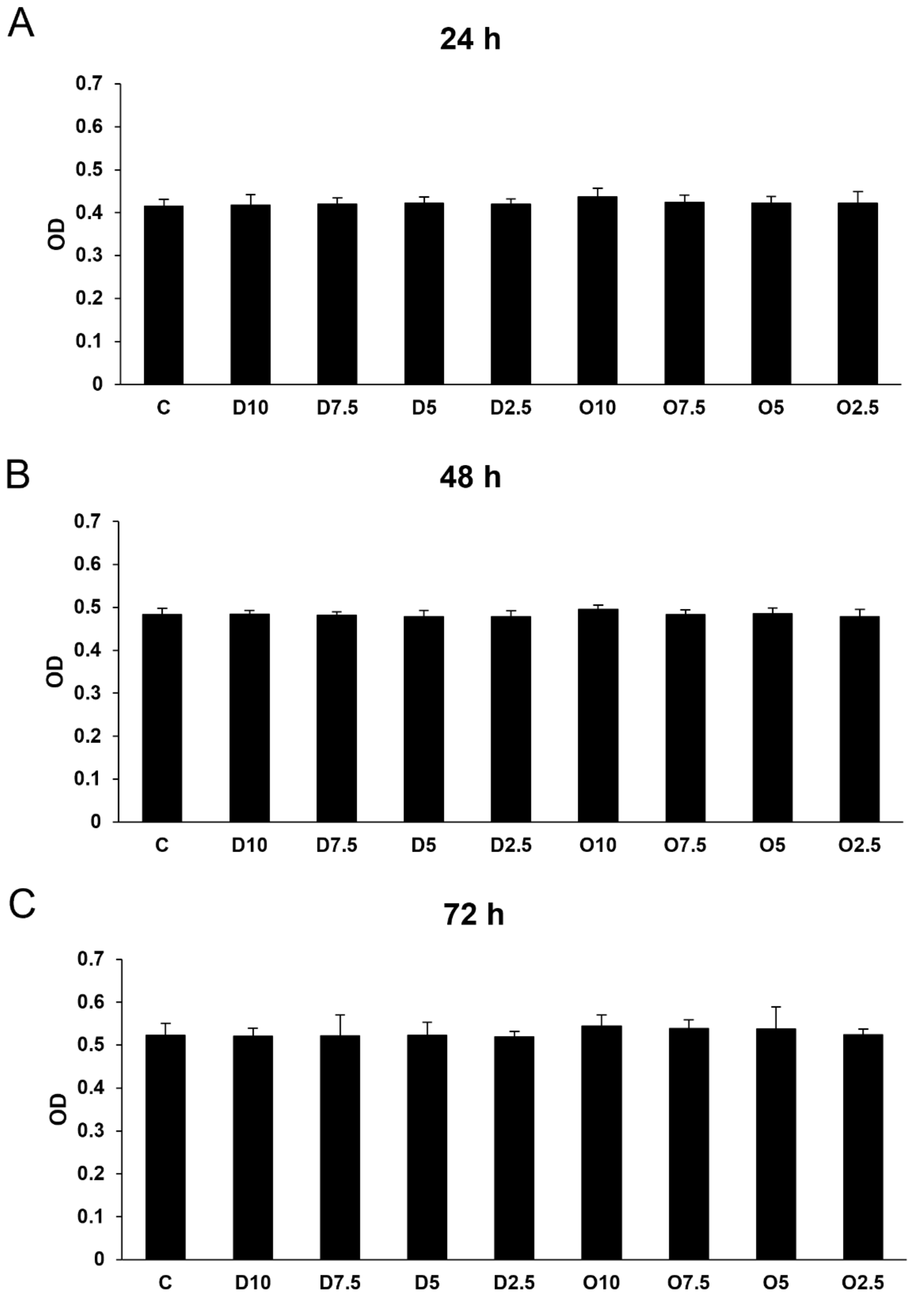

Figure S2. Cell viability determinations of the 3′-oxolutein-treated SH-SY5Y cells. Viability was measured using a resazurin-based cell viability assay after 3′-oxolutein treatments. The mean optical density of the wells indicates the viable cells from four independent experiments, each measured in triplicate. Viability is expressed as a percentile of the total cell number of the appropriate DMSO-treated control cells. (A) Cell viability of 3′-oxolutein and DMSO-treated SH-SY5Y cells at 24 h. (B) Cell viability of 3′-oxolutein and DMSO-treated SH-SY5Y cells at 48 h. (C) Cell viability of 3′-oxolutein and DMSO-treated SH-SY5Y cells at 72 h. The bars represent mean values and error bars represent the standard deviation (± SD) for four independent experiments (n=4). Abbreviations of treatments: C-absolute control; DMSO controls: D2.5-DMSO equivalent to 2.5 ng/μL of 3′-oxolutein; D5-DMSO equivalent to 5 ng/μL of 3′-oxolutein; D7.5-DMSO equivalent to 7.5 ng/μL of 3′-oxolutein; D10-DMSO equivalent to 10 ng/μL of 3′-oxolutein; O2.5- 2.5 ng/μL of 3′-oxolutein; O5- 5 ng/μL of 3′-oxolutein; O7.5- 7.5 ng/μL of 3′-oxolutein; O10- 10 ng/μL of 3′-oxolutein, OD-optical density.

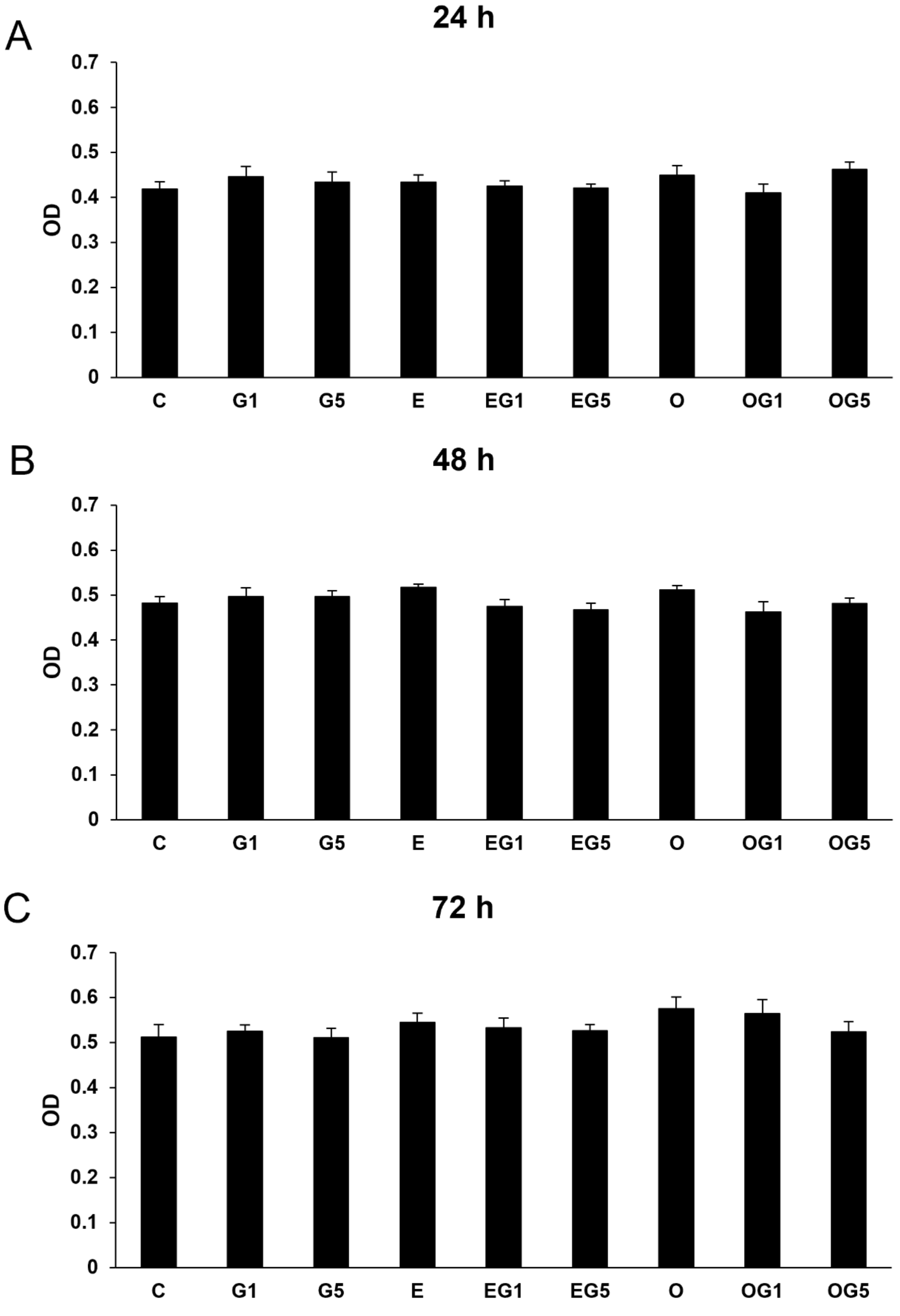

Figure S3. Cell viability of 3′-epilutein, 3′-oxolutein and glutamate-treated SH-SY5Y cells. Cell viability determinations of the 3′-oxolutein-treated SH-SY5Y cells. Viability was measured using a resazurin-based cell viability assay after 3′-oxolutein treatments. The mean optical density of the wells indicates the viable cells from four independent experiments, each measured in triplicate. Viability is expressed as the percentile of the total cell number of the untreated control cells, in the case of 3′-epilutein and 3′-oxolutein as a percentile of the total cell number of the appropriate DMSO-treated control cells. (A) Cell viability of 3′-epilutein, 3′-oxolutein and glutamate-treated SH-SY5Y cells at 24 h. (B) Cell viability of 3′-epilutein, 3′-oxolutein and glutamate-treated SH-SY5Y cells at 48 h. (C) Cell viability of 3′-epilutein, 3′-oxolutein and glutamate-treated SH-SY5Y cells at 72 h. The bars represent mean values and error bars represent the standard deviation (± SD) for four independent experiments (n = 4). Abbreviations: C- control; G1- 1mM glutamate; G5- 5 mM glutamate; E- 3′-epilutein; O- 3′-oxolutein; EG1- 3′-epilutein + 1 mM glutamate; EG5- 3′-epilutein + 5 mM glutamate; OG1- 3′-oxolutein + 1 mM glutamate; OG5- 3′-oxolutein + 5 mM glutamate; OD-optical density.
